# Case report: Treatment of constrictive epicarditis using the waffle procedure in a dog that had previously undergone a subtotal pericardiectomy

**DOI:** 10.3389/fvets.2024.1335433

**Published:** 2024-04-12

**Authors:** Rebecca Saunders, Steven Garnett, Brittany Lucchetti, Sophy Jesty

**Affiliations:** Charleston Veterinary Referral Center, Charleston, SC, United States

**Keywords:** pericardiectomy, waffle, epicarditis, constrictive, pericardial effusion

## Abstract

A 10 year-old female spayed German Short-haired Pointer dog weighing 26.8 kg (59 lb) presented with a 2 week history of recurrent ascites. The dog had a 4 year history of idiopathic pericardial effusion causing sporadic episodes of cardiac tamponade and secondary ascites. A subtotal pericardiectomy was performed 3 months prior to presentation. The patient had done well for 2 months following this procedure, at which point the large-volume modified transudate ascites recurred, necessitating abdominocentesis every 10 days. Thoracic and abdominal computed tomography (CT) revealed no abdominal or vascular cause of ascites. Transthoracic echocardiography performed under general anesthesia showed constrictive epicarditis (visceral pericarditis) resulting in diastolic dysfunction and right-sided congestive heart failure. A sternotomy was performed for a pericardial waffle procedure or crosshatch pericardiotomy—scoring of crosshatched incisions into the thickened epicardium. Echocardiographic findings postoperatively were consistent with resolved constrictive epicarditis. At 8 months postoperatively, the dog was clinically normal and had only required one abdominocentesis one month after the waffle procedure. This case report describes the successful treatment of a dog with constrictive epicarditis using a novel surgical technique (waffle procedure) that has not yet been described in veterinary medicine.

## Introduction

In dogs, pericardial diseases causing pericardial effusion result in signs of right-sided heart failure or cardiac tamponade as a result of ventricular compression and restriction of diastolic ventricular volume (1). Constrictive pericarditis (CP) is a rare pericardial disorder, often of unknown etiology, that is challenging to diagnose in both human and veterinary patients without invasive and costly cardiac catheterization (2). On echocardiogram, CP may be characterized by a thickened pericardium (not always present), small volume pericardial effusion, and intracardiac hemodynamic abnormalities secondary to loss of pericardial compliance (3, 4).

Treatment of choice for constrictive pericarditis in both people and animals is a parietal pericardiectomy (5). In rare cases of pericardial constriction in humans, the epicardium (i.e., visceral pericardium) is also thickened and calcified such that a conventional pericardiectomy does not alleviate the constrictive physiology and, therefore, clinical signs persist. In these cases, a waffle procedure is performed to alleviate the epicardial scarring by creating crosshatching incisions across the entire surface of both ventricles, thus relieving constriction and allowing for appropriate ventricular filling (5, 6).

This case report is the first in veterinary medicine to detail the diagnosis of constrictive epicarditis in a dog that had previously undergone a subtotal pericardiectomy (removal of the parietal pericardium) as well as successful treatment with a surgical technique never previously reported in the dog.

## Patient information

Informed consent was obtained from the owner for publication of this case report. A 10 year-old female spayed German Short-Haired Pointer dog weighing 26.8 kg was presented to Charleston Veterinary Referral Center for recurrent ascites. The dog had a 4 year history of idiopathic pericardial effusion resulting in 4 episodes of cardiac tamponade with ascites occurring between 6 and 18 months apart. Three months prior to presentation, the patient underwent subtotal pericardiectomy and recovered well. Histopathology of the harvested pericardium revealed severe diffuse chronic fibrosing pericarditis with no infectious agents or neoplastic cells identified.

## Clinical findings

On physical examination at presentation, the dog had normal vitals (heart rate 100 beats/min; temperature 101.0 F; respiratory rate 24 breaths/min), normal heart sounds, synchronous pulses and severe abdominal distention with a ballotable fluid wave and jugular pulses. Blood tests showed no abnormal findings. Thoracic and abdominal CT confirmed the presence of peritoneal effusion with unknown etiology, normal cardiovascular structures and no evidence of pulmonary metastatic disease or intravascular thrombi. The ascites was characterized as a modified transudate on cytology.

## Diagnostic assessment

**Figure 1:**
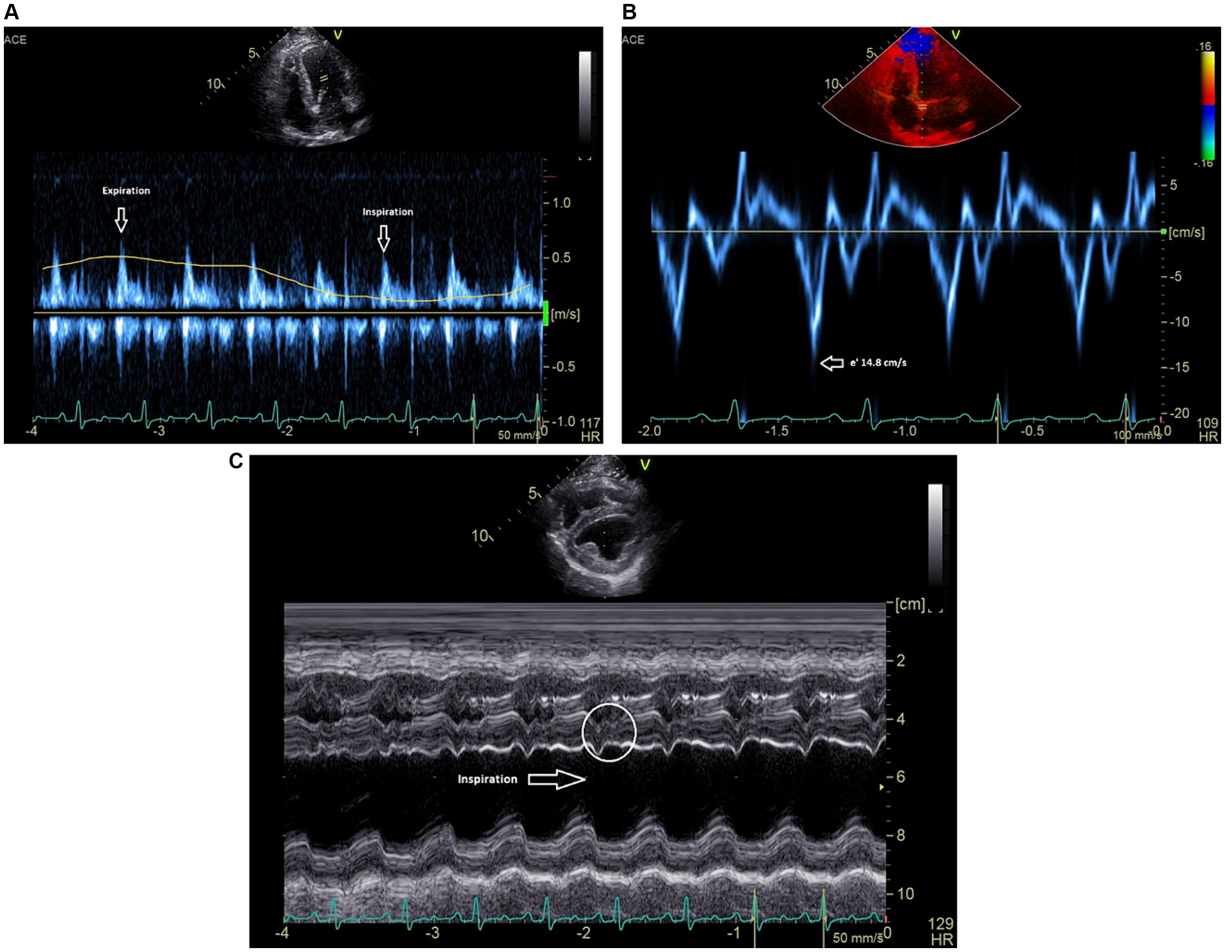
**(A)** Pulsed-wave Doppler spectrum of mitral inflow velocities demonstrates a marked respiratory variation of the peak E-wave velocities. **(B)** Medial mitral annulus early diastolic (e’) tissue Doppler velocities near 15 cm/s. **(C)** M-mode recording (short axis) showing leftward ventricular septal shift in inspiration. Beat-to-beat septal diastolic shudder is also noted.

All two-dimensional echocardiographic images were obtained using a 5 MHz phased array transducer (GE Vivid E90). On echocardiography, constrictive epicarditis was confirmed based on the following key findings: respiratory variation of >25% in the mitral E-wave velocity during respiration (Figure 1A); significant respiratory variation on diastolic tricuspid inflows; a septal mitral annular e’ velocity of ≥15 cm/s (Figure 1B); moderate diastolic reversal of the hepatic venous flow with expiration; and leftward ventricular septal shift with inspiration on M-mode imaging of the ventricular septum (Figure 1C) (5, 7). Based on this diagnosis, it was recommended that any remaining parietal pericardial tissue be surgically removed and, more importantly, epicardial excision be concurrently performed to relieve epicardial constriction and restore diastolic function. Because of the promising published results from the waffle procedure in human medicine and the paucity of veterinary studies citing the manner by which to perform epicardial excision in dogs, it was elected to perform a waffle procedure in this patient.

## Therapeutic intervention

The patient was sedated with oral Trazodone (3.5 mg/kg every 8 h) on the 2 days preceding and the morning of anesthesia and premedicated with intravenous hydromorphone (0.1 mg/kg) and midazolam (2 mg/kg). Anesthesia was induced with propofol to effect (12 mL given) and maintained with vaporized isoflurane which did not exceed 1.5% during the procedure, a continuous rate infusion of lidocaine (50 mcg/kg/min) to mitigate ventricular arrhythmias that may occur with cardiac manipulation, and a continuous rate infusion of ketamine (0.5 mg/kg/h) and fentanyl (10 mcg/kg/h). Intravenous fluids were administered at a rate of 5 mL/kg/h. The patient was placed in dorsal recumbency followed by aseptic preparation of the ventral thorax and abdomen. Mechanical ventilation was started prior to incision of the thoracic wall. A ventral midline thoracic incision was made followed by sternotomy using a gas-powered sagittal saw. Thoracic explore revealed adherence of the remaining parietal pericardium to the dorsal and cranial aspects of the heart and a firm, fibrous epicardium. The remaining parietal pericardium was dissected away from the heart using both blunt and Ligasure^™^ dissection and excised to the level of the phrenic nerves. During this phase of the procedure, the patient became hemodynamically compromised, experiencing hypotension (direct arterial pressure monitoring measuring 60/30 mmHg) and profound sinus bradycardia and 2nd degree AV block (heart rate 30–40 bpm). The patient was taken off gas anesthesia and maintained on total intravenous anesthesia, via increased doses of ketamine (1 mg/kg/h) and fentanyl (20 mcg/kg/h). A norepinephrine constant rate infusion via a new peripheral intravenous catheter was added and maintained at a rate of 0.2–0.6 mcg/kg/min. Two doses of intravenous atropine were also administered (0.02 mg/kg). This resulted in slight improvement to the patient’s hemodynamic parameters characterized by an invasive blood pressure of 70/30 mmHg and heart rate of 60 bpm. The arterial catheter experienced significant overdamping artifact intermittently relieved by flushing throughout the procedure.

**Figure 2:**
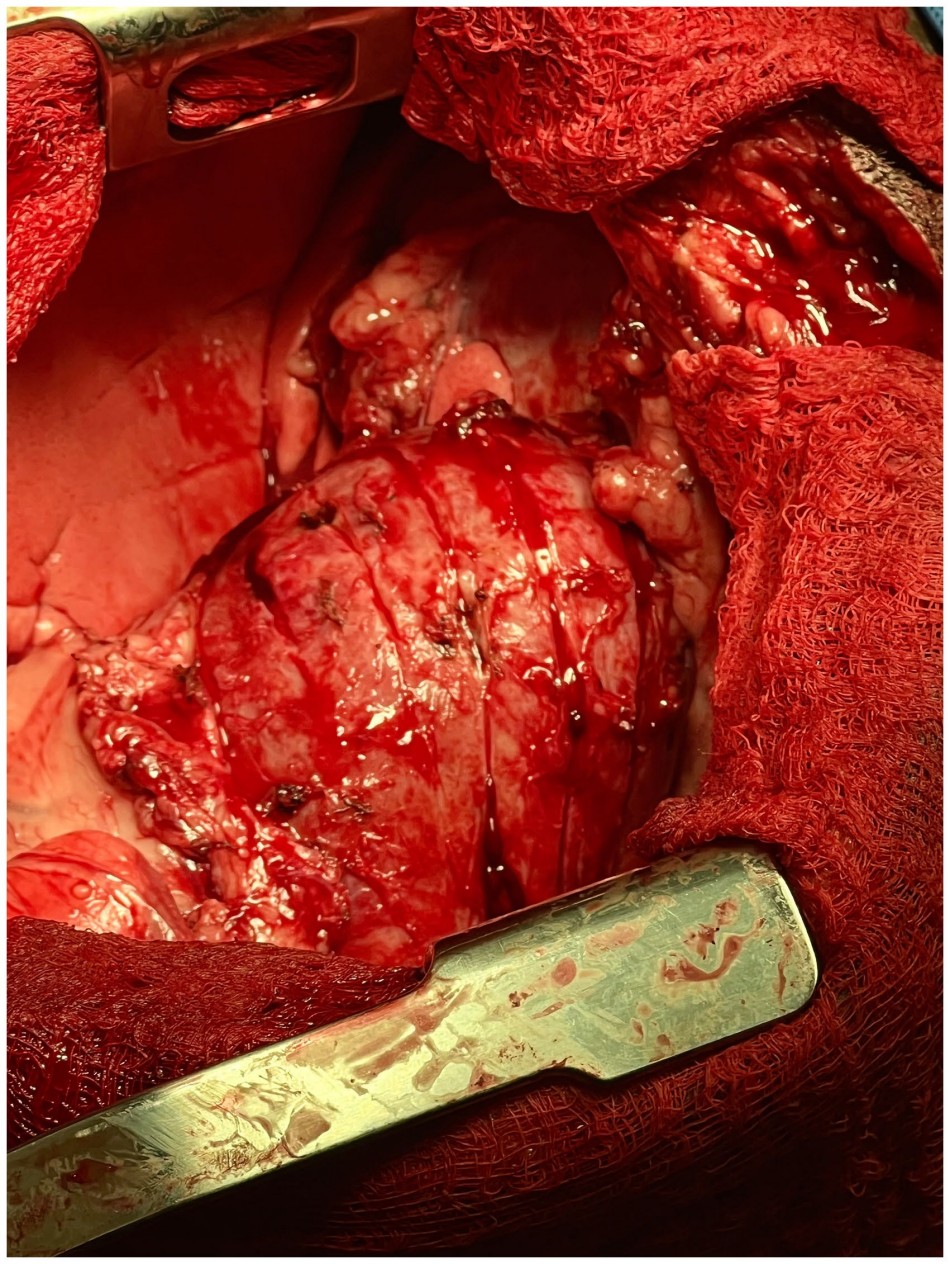
Intra-operative photograph of the longitudinal epicardial incisions made into the left ventricular wall of the heart in the canine patient described in this report.

Next, using a #10 scalpel and curved iris scissors, partial thickness incisions were made in a grid-like pattern through the left ventricular wall followed by the right ventricular wall. The longitudinal incisions were made first followed by the crosshatching (Figure 2). The incisions were approximately 1 cm apart and 5 mm deep. Deep dissection was continued until the tension on the epicardium was relieved and the sides of the crosshatches separated. As soon as two longitudinal incisions were made into the epicardium, the patient’s blood pressure normalized to 90/50 mmHg and heart rate improved to 110 bpm indicating improved hemodynamics. Mild hemorrhage was controlled with bipolar cautery. Subjectively, the left ventricle had more expansion and improved contraction following the formation of the waffle. A left-sided 12 Fr 30 cm MILA chest tube was placed and secured with nylon. The thoracic cavity was lavaged routinely with saline. The sternotomy incision was apposed using 1 PDS in a cruciate pattern. Intrathoracic muscles were apposed using 0 PDS in a simple continuous pattern. Nocita^®^ (5.3 mg/kg) was injected intramuscularly bilaterally along the incision. The subcutaneous space was closed using 3-0 Monocryl in a simple continuous pattern followed by an intradermal closure with 3-0 Monocryl in a simple continuous pattern. Skin staples were then applied. Using the chest tube, the thorax was evacuated of any remaining air and fluid. The total anesthetic time was 3 h and 10 min with a surgical time of 2 h and 15 min.

**Figure 3:**
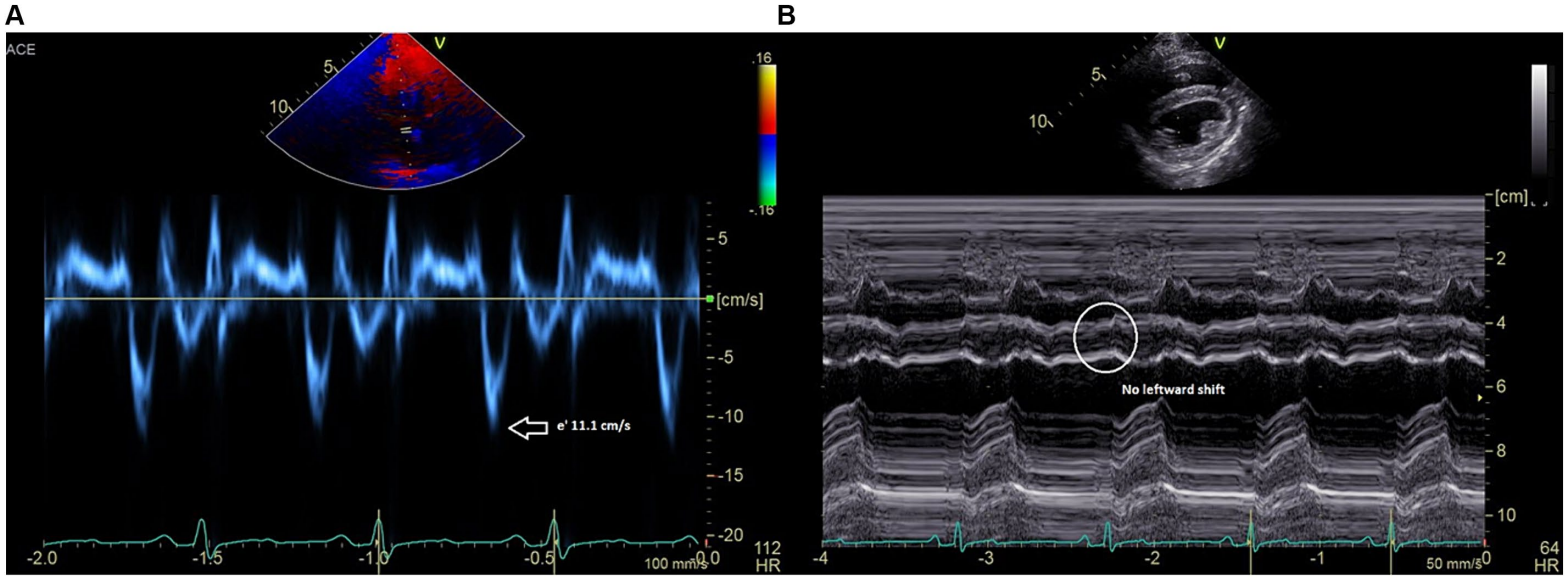
**(A)** Medial mitral annulus early diastolic (e’) tissue Doppler velocities are reduced to 11 cm/s. **(B)** M-mode recording (short axis) showing resolved leftward ventricular septal shift and resolved beat-to-beat septal diastolic shudder.

Post-op echocardiography performed under the same anesthetic event revealed a normalized mitral and tricuspid inflow pattern with respiration, reduced septal mitral annular e’ velocity of ~11.1 cm/s (Figure 3A), and no hepatic venous flow reversal during expiration. Left ventricular internal diastolic dimensions and estimates of systolic function were also improved (LVIDd 33.7 compared to pre-waffle which measured 29.0; FS 40% compared to pre-waffle which measured 25%) and no leftward septal shift was noted on inspiration (Figure 3B). The patient recovered uneventfully from anesthesia. Mild ventricular arrhythmias were appreciated on telemetry a few hours postoperatively and resolved with a single intravenous bolus of magnesium chloride (3 mEq/kg) diluted 1:3 with 0.9% NaCl and given over 15 min.

## Patient outcome

Histopathology performed on harvested epicardial tissue showed marked fibrosis with multifocal necrosis and lymphocytic, histiocytic and neutrophilic endocarditis. There was no evidence of infectious disease or neoplasia.

The dog was discharged 4 days postoperatively on oral Gabapentin (10 mg/kg) every 8–12 h with no apparent complications and no recurrent ascites. Once biopsy results were obtained, Spironolactone (2 mg/kg twice daily) was initiated for the antifibrotic benefits. A single abdominocentesis was required 4 weeks post-operatively. Since that abdominocentesis was performed, 7 months have elapsed and there has been no significant accumulation of ascites.

## Discussion

CP is rare in both humans and veterinary species. There are limited case reports in cats, cattle and horses (8–11). A small case series in which the cardiac catheterization hemodynamics of constrictive pericarditis was described in dogs was published in 1984 (1) as well as a few reports and a series of 17 cases of coccidioidomycosis-induced effusive-constrictive pericarditis in canines published in 2005 (4, 12, 13). The cause of CP is often unknown, but when identified in animals, it can be caused by metallic foreign bodies, infectious agents such as actinomycosis and coccidioidomycosis, chronic conditions such as chylothorax or idiopathic pericardial effusion and neoplasms such as lymphoma, mesothelioma or carcinomatosis of the pericardium (2, 8, 14). In humans, the most common causes are idiopathic (61%) and following cardiac surgery (37%) (15).

### Diagnosis

The chief hemodynamic consequence of CP is markedly reduced ventricular filling and, therefore, ventricular volume with enhanced interventricular dependence as a result of encasement of the heart within a noncompliant fibrotic membrane. Although the constriction affects both ventricles equally, the clinical syndrome is characterized by signs of right-sided heart failure due to elevation of central venous pressure (1, 8).

Invasive hemodynamic pressure measurement via cardiac catheterization is the gold standard for diagnosing CP in humans. Hallmark findings include elevation and equalization of end-diastolic pressures in both ventricles (ventricular interdependence) and a “square root” or early diastolic dip and mid-diastolic plateau sign on the ventricular tracings with prominent *x* and *y* descents on atrial tracings (1). Due to the cost and invasive nature of this diagnostic test, cardiac catheterization is often not performed in veterinary patients.

In this case, echocardiographic findings were used to strongly support a diagnosis of constrictive visceral epicarditis. In the heart with a normal pericardium, the left and right ventricles can fill relatively independently such that during inspiration, when negative intrathoracic pressure increases venous return to the right heart, the right ventricular free wall can move eccentrically to allow for increased filling (8, 16). With CP, however, the increased right ventricular filling with inspiration occurs with compromise to the left ventricle due to enhanced ventricular interdependence. The ventricular septum is forced leftward, which can be seen on M-mode in this patient (Figure 1C). Due to compromised left-sided filling during inspiration, respiratory variation of >25% in the mitral E-wave velocity is considered to be a specific sign of constrictive pericarditis in human patients (7, 16) and was documented in this case (Figure 1A). Finally, a hepatic vein expiratory diastolic reversal ratio >/= 0.79 has a 87% sensitivity and 91% specificity for diagnosing constrictive pericarditis in humans, and was observed in this as well (16). All echocardiographic features of constrictive pericarditis were resolved in this case following the waffle procedure.

### Surgical treatment

In both veterinary and human patients, pericardiectomy is the standard procedure performed to relieve the diastolic dysfunction caused by constrictive pericarditis. It is associated with a mortality rate of 6–19% in human studies (17). In a small subset of human patients with constrictive pericarditis, the epicardium is also thickened, resulting in constrictive epicarditis. These affected patients do not improve postoperatively, requiring a repeat surgery called the waffle procedure (5, 15).

**Figure 4:**
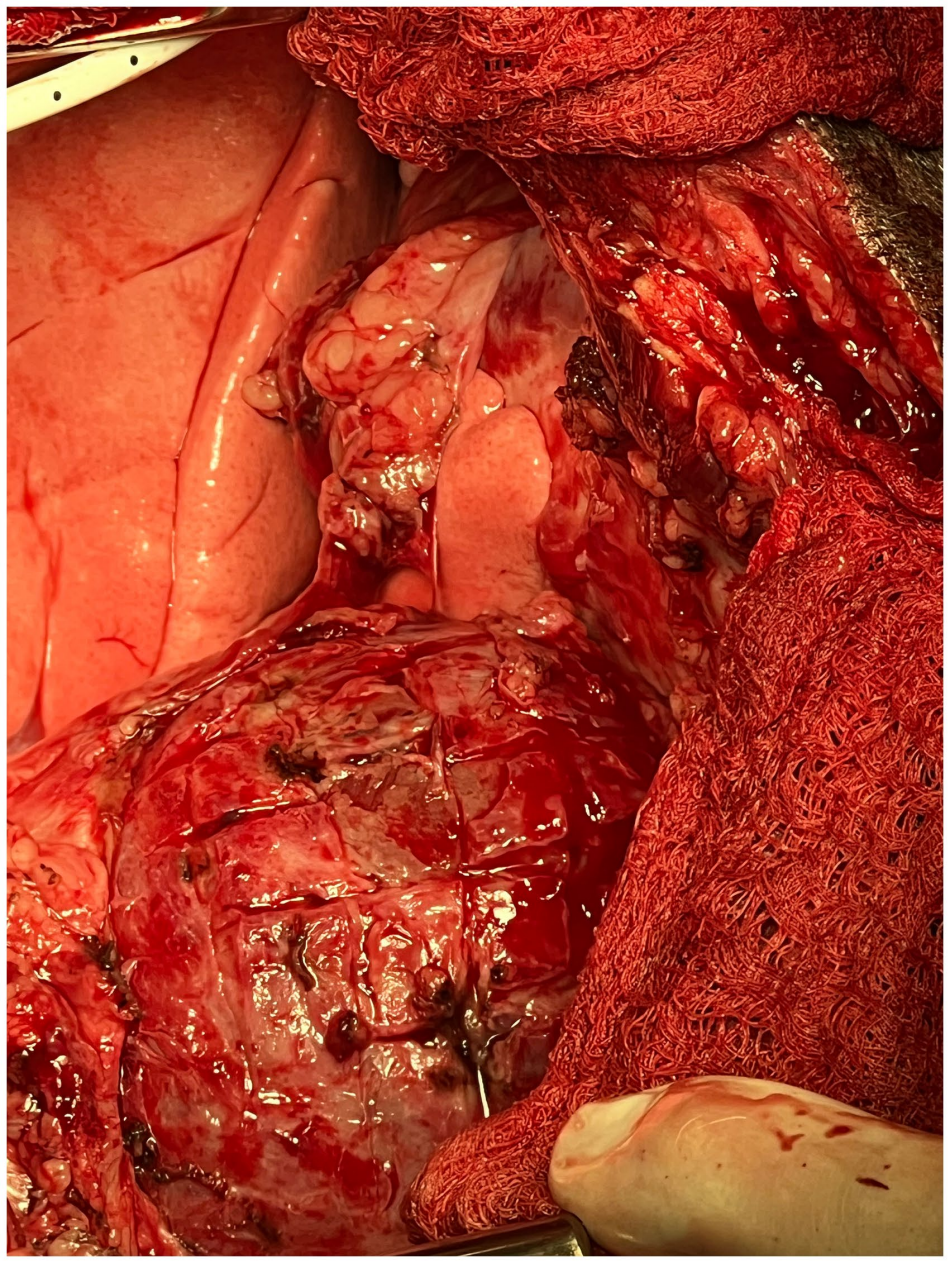
Intra-operative photograph of the completed epicardial waffle in the canine patient described in this report.

The waffle procedure was first described in human medicine in 1983 by Heimbecker as a less traumatic, effective alternative to epicardial resection in patients with constrictive epicarditis. After the parietal pericardium was removed, the technique was described as a series of epicardial incisions made with electric and/or sharp dissection in both the longitudinal and transverse directions (grid-like). A grooved director was then advanced under the thickened epicardium to peel away the constrictive tissue as atraumatically as possible (18). The entire surface of both ventricles was incised in a similar manner until the epicardium had been converted into a waffle-like pattern of 1 cm^2^ islands of scar tissue (Figure 4). Of note, it is imperative to begin the procedure over the left ventricle to avoid acute left heart failure. If a waffle incision is made first over the right ventricle, the left ventricle would immediately suffer from massive preload without the “pop-off” of diastolic dysfunction, resulting in acute fulminant pulmonary edema (19). Once the waffle is complete, immediate ventricular expansion and increased contractility are often observed.

Since this technique was originally described in 1983, there have been 10 other articles referencing the waffle procedure in human medicine with short-term successful outcomes (20). Although there is a report of epicardial excision or stripping in dogs (13), to the author’s knowledge, this report is the first description of the waffle procedure in veterinary medicine. Even more uniquely, it was performed in a patient that had previously undergone subtotal pericardiectomy and had recurrent right-sided congestive heart failure signs months later.

### Outcome

Human studies have shown that the waffle procedure, performed in conjunction with a total pericardiectomy for constrictive pericarditis with epicardial thickening, resulted in superior postoperative diastolic function compared to total pericardiectomy alone (6, 20). Additionally, adding the waffle procedure to total pericardiectomy did not significantly increase operative time, blood loss, perioperative blood transfusion or post-operative stay in these patients (6). There are no reports in the human literature about long-term survival for patients undergoing the waffle procedure compared to those undergoing pericardiectomy alone. A retrospective study by Gatti et al. included 81 human patients that underwent pericardiectomy for constrictive pericarditis, including the waffle procedure in several of these patients. It showed a 10 year survival free of all-cause death of 76.9% with complete removal of cardiac constriction through epicardial excision potentially enhancing long-term outcomes (21). The veterinary case series by Heinritz et al. detailing outcomes of 17 dogs with coccidioidomycosis-induced effusive-constrictive pericarditis treated with subtotal pericardiectomy and epicardial excision showed a perioperative mortality rate of 23.5% and 2 year post-discharge survival rate of 82% (13).

In conclusion, this case report describes the echocardiographic diagnosis of constrictive epicarditis and successful surgical treatment with a waffle procedure in a patient having previously undergone subtotal pericardiectomy. At the time of this writing, 8 months post-waffle, the patient remains asymptomatic. Additional cases are required to validate the safety and efficacy of the waffle procedure for this uncommon condition in veterinary patients, but this report shows encouraging results.

## Data availability statement

The original contributions presented in the study are included in the article/supplementary material, further inquiries can be directed to the corresponding author.

## Author contributions

RS: Writing – review & editing, Writing – original draft. SG: Writing – review & editing. BL: Writing – review & editing. SJ: Writing – review & editing.
